# Is the 2013 American Thoracic Society CPAP-tracking system algorithm useful for managing non-adherence in long-term CPAP-treated patients?

**DOI:** 10.1186/s12931-019-1150-7

**Published:** 2019-09-12

**Authors:** Marie-Caroline Rotty, Jean-Pierre Mallet, Carey M. Suehs, Christian Martinez, Jean-Christian Borel, Claudio Rabec, Arnaud Bourdin, Nicolas Molinari, Dany Jaffuel

**Affiliations:** 1 IMAG, CNRS, Montpellier University, Montpellier University Hospital, Montpellier, France; 2 Apard groupe Adène, Montpellier, France; 3 Department of Respiratory Diseases, Montpellier University Hospital, Arnaud de Villeneuve Hospital, 371, Avenue Doyen Giraud, 34295 Montpellier Cedex 5, France; 4 Department of Medical Information, Montpellier University Hospital, Montpellier, France; 5 Grenoble Alps University, Inserm U1042, HP2 (Hypoxia PhysioPathology) Laboratory, Centre Hospitalier Universitaire Grenoble Alpes, Grenoble, France; 6 Pulmonary Department and Respiratory Critical Care Unit, University Hospital Dijon, Dijon, France; 7 PhyMedExp (INSERM U 1046, CNRS UMR9214), Montpellier University, Montpellier, France; 8 Pulmonary Disorders and Respiratory Sleep Disorders Unit, Polyclinic Saint-Privat, Boujan sur Libron, France

**Keywords:** CPAP, Leaks, Apnea-hypopnea index, Telemedicine

## Abstract

**Background:**

Whereas telemedicine usage is growing, the only clinical algorithm for Continuous Positive Airway Pressure (CPAP) adherence management is that stipulated by the 2013 American Thoracic Society (ATS). The capacity of the latter to predict non-adherence in long-term CPAP-treated patients has not been validated.

**Methods:**

Patients from the prospective real-life InterfaceVent study (NCT03013283, study conducted in an adult cohort undergoing at least 3 months of CPAP) and eligible for ATS algorithm usage were analysed. The residual device Apnea–Hypopnea-Index (AHI_flow_) and High Large Leak (HLL) thresholds proposed in the ATS algorithm were evaluated for predicting adherence (i.e. AHI_flow_ > 10/h, HLLs 95th > 24 L/min for ResMed® devices and ResMed® nasal mask, HLLs 95th > 36 l/min for ResMed® devices and ResMed® oronasal masks, HLLs > 1 h for Philips® devices and HHLs > 60 l/min for Fisher & Paykel® devices). Adherence was defined according to the 2013 ATS algorithm (i.e. CPAP use > 4 h/j for at least 70% of days).

**Results:**

650/1484 patients eligible for ATS algorithm usage were analysed (15.38% non-adherent, 74% male with a median (IQ_25–75_) age of 68 (61–77) years, a body mass index of 30.8 (27.7–34.5) kg/m^2^, an initial AHI of 39 (31–55) events/h, and CPAP-treatment-duration of 5.1 (2.2–7.8) years). Logistic regression analysis demonstrated no significant relationship between the ATS proposed AHI_flow_ or HLL thresholds and non-adherence. Complementary ROC curve analysis failed to determine satisfactory AHI_flow_ and HLL thresholds.

**Conclusion:**

When managing non-adherence in long-term CPAP-treated patients, our data do not validate absolute AHI_flow_ or HLL thresholds in general.

**Trial registration:**

The INTERFACE-VENT study is registered on ClinicalTrials.gov (Identifier: study (NCT03013283).

## Background

Continuous Positive Airway Pressure (CPAP) is the cornerstone of obstructive sleep apnea treatment. Previous studies have reported a correlation between patient adherence and treatment outcomes [1]. CPAP devices can track adherence, but also leaks and residual Apnea–Hypopnea-Index (AHI_flow_) values. CPAP tracking systems intuitively seem useful, but there are few data demonstrating the benefit of such systems in improving CPAP adherence [2]. In particular, the clinical significance of device-reported leaks or device reported AHI_flow_ is unknown. As underlined in the 2013 American Thoracic Society (ATS) statement, it was speculated that High Large Leaks (HLLs) and high AHI_flow_ may affect CPAP adherence. Thus, HLLs and AHI_flow_ reported by manufacturers were cautiously included in the 2013 ATS clinical algorithm for using CPAP adherence tracking systems [3]. This statement is the only one available to clinicians, and despite increasing telemedicine usage in the field, it remains untested. In this context, the aim of this study is to assess the impact of HLLs and high AHI_flow_ on the adherence of long-term CPAP-treated patients in real-life conditions.

## Methods

### Study design

**Fig. 1:**
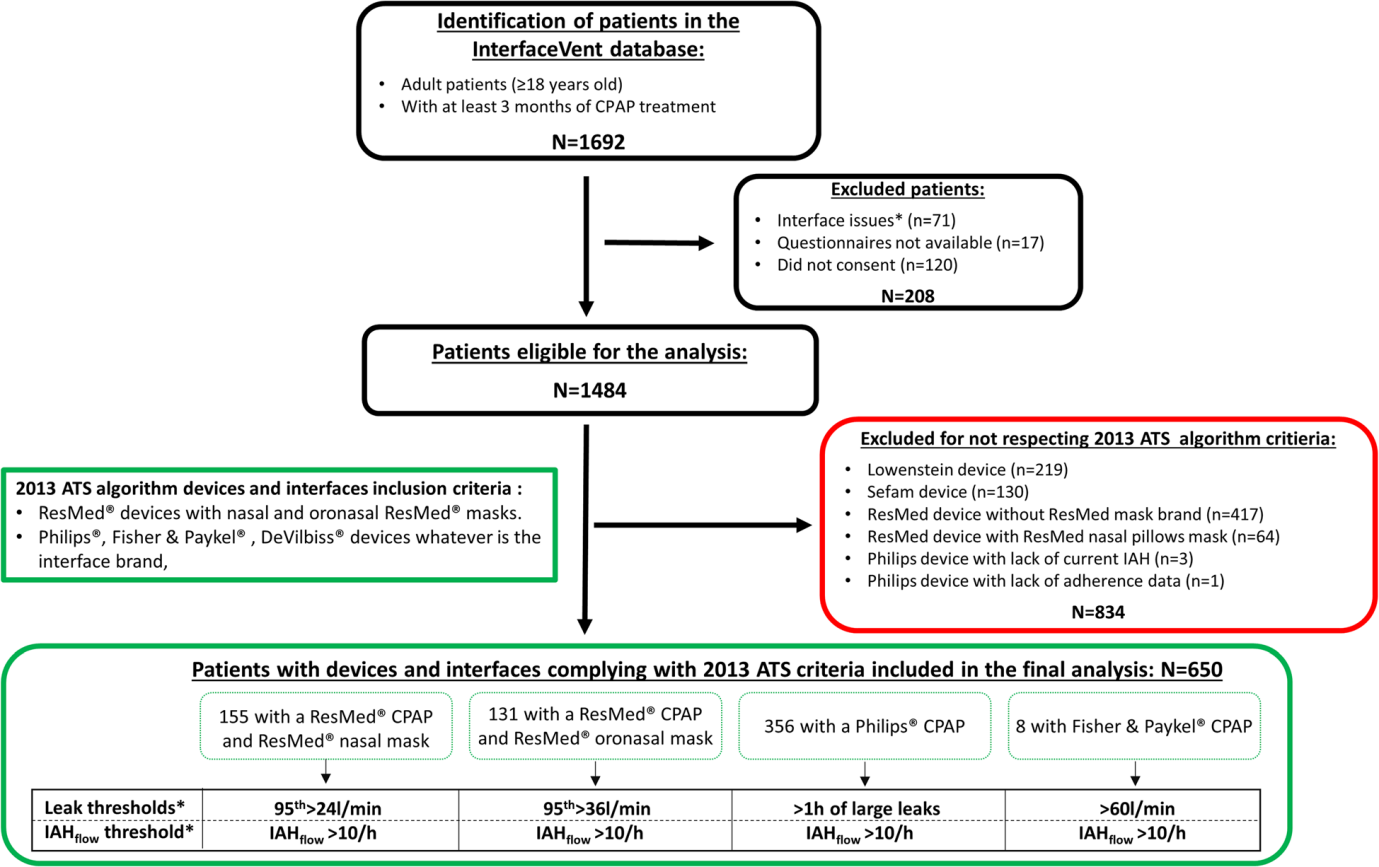
The study flow chart. Patients in the InterfaceVent study (NCT03013283) meeting 2013 ATS algorithm criteria and lacking interface or data availability problems were included in the final analysis. The four subgroups correspond to different device-mask combinations and their associated thresholds* as foreseen in the ATS criteria

The InterfaceVent study is a prospective real-life study conducted in an adult cohort undergoing at least 3 months of CPAP for sleep apnea syndrome (SAS), defined according to the French Social Security system criteria: 1) Apnea Hypopnea Index (AHI) ≥ 30/h (or AHI ≥ 15/h and more than 10/h respiratory-effort-related arousal), and 2) associated with sleepiness and > 3 symptoms from among snoring, headaches, hypertension, reduced vigilance, libido disorders, nycturia). Following an initial prescription by one of the 336 device-prescribing physicians in the *Occitanie* region of France, these patients were provided care by the association “Apard”, ADENE group, a non-profit home-care provider. Patient inclusions were performed during a home-visit by one of the 32 Apard technicians. Patients included in the analysis are those for whom it is possible to apply the 2013 ATS clinical algorithm for using CPAP adherence tracking systems (Fig. 1).

### ATS algorithm thresholds

The AHI_flow_ threshold and HLLs thresholds chosen in the present paper are those proposed in the 2013 ATS algorithm (i.e. AHI_flow_ > 10/h, HLL 95th > 24 L/min for ResMed® devices and ResMed® nasal mask, HLL 95th > 36 l/min for ResMed® devices and ResMed® oronasal masks, HLL > 1 h of large leaks for Philips® devices and HHL >  60 l/min for Fisher & Paykel® devices whatever the type and brand of the interface). Adherence was also defined according to the 2013 algorithm (CPAP use > 4 h/j at least 70% of days).

### Collected data

In addition to demographic/clinical data and the mask/device data, the response to the Epworth Sleepiness Scale (ESS) and the Euroqol 5 Dimensions 3 level version (EQ-5D-3 L) questionnaires were also collected. Patient perceptions of leaks and mouth dryness were assessed using an 11-point visual analogue scale (VAS) (ranging from no discomfort (0) to very uncomfortable (10)).

### Statistical analyses

Multivariable logistic regression analyses were used to study associations between adherence and collected data. Using forward-stepwise selection, covariates with a univariable *p*-value < 0.15 were fed into multivariable analyses. Odds-ratios with their 95% CI were calculated according to Woolf’s method, with an alpha risk of 0.05. Model goodness-of-fit was assessed using the Hosmer-Lemeshow test. Receiver operating curves (ROC) were created to determine AHI_flow_ and HLL thresholds predictive of non-adherence, as determined by maximizing the Youden index.

## Results

Six hundred fifty patients were analysed: 155 with a ResMed® CPAP and ResMed® nasal mask, 131 with a ResMed® device and ResMed® oronasal mask, 356 with a Philips® device regardless of the interface used and 8 with Fisher & Paykel Device. The results of the logistic regression analysis with adherence as the dependent variable are summarized in Table 1.

**Table 1:** Logistic regression analysis with adherence (> 4 h /day, 70% of the days) as the dependent variable

	Whole population	Non adherent *N* = 100	Adherent *N* = 550	Crude OR [95% CI]	*p*-value	Adjusted OR [95% CI]	*p*-value
Demographics							
Age (yrs)	68 [61; 74]	68 [60; 75]	68 [61; 74]	1.00 [0.98; 1.02]	0.80		
Gender, female (%)	173/650 (26.6)	32/100 (32.0)	141/550 (25.6)	0.73 [0.46; 1.16]	0.19		
BMI (kg/m^2^)	30.8 [27.7; 34.5]	29.4 [26.5; 32.3]	31.1 [27.8; 34.9]	1.07 [1.02; 1.12]	0.003	1.07 [1.01; 1.13]	0.03
Initial AHI (event/h)	39 [31; 55]	37.9 [30.0; 52.0]	39.0 [31.0; 57.0]	1.01 [0.99; 1.02]	0.16		
Active smokers (%)	77/637 (12.1]	13/99 (13.1)	64/538 (11.9)	0.89 [0.47; 1.69]	0.73		
Beard (%)	75/456 (16.5)	10/64 (15.6)	65/392 (16.6)	1.05 [0.50; 2.18]	0.70		
Mustache (%)	42/456 (9.2)	7/64 (10.9)	35/392 (8.9)	0.81 [0.34; 1.92]	0.60		
No mustache no beard (%)	339/456 (74.3)	47/64 (73.4)	292/392 (74.5)	Ref	0.88		
Presence of partner	457/640 (71.4)	61/96 (63.5)	396/544 (72.8)	1.54 [0.97; 2.42]	0.07	2.03 [1.18; 3.50]	0.011
Active workers	130/632 (20.6)	20/95 (21.1)	110/537 (20.5)	0.97 [0.57; 1.65]	0.90		
ESS (VAS score)	5 [3; 8]	6 [3; 8]	5 [3; 9]	0.99 [0.95; 1.05]	0.87		
EQ-5D-3 L							
Problems with mobility (%)	157/623 (25.2)	25/94 (26.6)	132/529 (26.6)	0.92 [0.58; 1.51]	0.74		
Problems with self-care (%)	38/617 (6.2)	6/94 (6.38)	32/523 (6.12)	0.96 [0.39; 2.35]	0.92		
Problems with usual activities (%)	124/620 (20.0)	20/96 (20.8)	104/524 (19.9)	0.94 [0.55; 1.61]	0.82		
Problems of pain/discomfort (%)	347/623 (55.7)	54/96 (56.3)	293/527 (55.6)	0.97 [0.63; 1.51]	0.91		
Problems of anxiety/depression (%)	240/624 (38.5)	41/96 (42.7)	199/528 (37.9)	0.81 [0.52; 1.26]	0.35		
EQ-5D-3 L Health (VAS score)	69.8 [50.2; 80.1]	69.2 [49.4; 80.0]	69.9 [50.4; 80.2]	1.01 [0.99; 1.02]	0.17		
Device							
Treatment duration (yrs)	4.9 [2.1; 9.8]	3.8 [1.3; 8.1]	5.1 [2.3; 10.2]	1.06 [1.01; 1.11]	0.02		
Fixed pressure (%)	91/650 (14.0)	12/100 (12.0)	79/550 (14.4)	1.23 [0.64; 2.35]	0.53		
90th/95th Pressure (cmH_2_O)	10.0 [8.30; 11.8]	9.9 [8.0; 11.5]	10.0 [8.3; 11.8]	1.10 [0.99; 1.21]	0.09		
Oronasal (%)	216/650 (33.2)	43/100 (43.0)	173/550 (31.5)	0.58 [0.37; 0.91]	0.14		
Nasal (%)	375/650 (57.7)	47/100 (47.0)	328/550 (59.6)	Ref	0.07		
Nasal pillows (%)	59/650 (9.1)	10/100 (10.0)	49/550 (8.91)	0.70 [0.33; 1.48]	0.83		
Heated humidifier	386/650 (59.4)	66/100 (66.0)	320/550 (58.2)	0.72 [0.46; 1.12]	0.14		
Heated breathing tube	22/650 (3.4)	3/100 (3.0)	19/550 (3.5)	1.16 [0.34; 3.99]	0.82		
2013 ATS statement tested thresholds							
Current AHI_flow_ (> 10)	23/650 (3.4)	5/100 (5.0)	17/550 (3.1)	0.61 [0.22; 1.68]	0.34		
ResMed Nasal large leaks (95th > 24 L)	53/154 (34.4)	10/22 (45.5)	43/132 (32.6)	0.58 [0.23; 1.45]	0.24		
ResMed Facial large leaks (95th > 36 L)	28/131 (21.4)	8/23 (34.8)	20/108 (18.5)	0.43 [0.16; 1.14]	0.09		
Philips leaks (> 1 h of large leaks)	11/356 (3.1)	1/55 (1.82)	10/301 (3.32)	1.86 [0.23; 14.8]	0.56		
Fisher Paykel leaks (> 60 L/min)	8/8 (100)	0/8 (0)	8/8 (100)	NA			
VAS scores							
Patient perceived leaks (VAS score)	3 [1; 5]	3.0 [1.0; 6.0]	3.0 [0.0; 5.0]	0.97 [0.91; 1.05]	0.48		
Patient perceived mouth dryness (VAS score)	3 [0; 7]	5 [1; 8]	3 [0; 7]	0.93 [0.88; 0.99]	0.019		

Univariable analysis failed to demonstrate a significant HLL or AHI_flow_ effect on adherence (Table 1). Interestingly, the VAS for leaks was not associated with non-adherence in the univariable analysis, whereas mouth dryness was (*p* = 0.019). Finally, multivariable logistic regression demonstrated that increased body mass index or the presence of a partner was significantly positively associated with adherence.

In order to re-evaluate AHI_flow_ and HLL thresholds associated with non-adherence, we generated ROCs curves. For ResMed®-reported AHI_flow_, accuracy was low (area under curve (AUC) of 0.53 [0.44–0.62]) for a threshold of 13.3/h (sensitivity was 0.04 and specificity was 0.99, positive predictive value (PPV) was 0.50 and negative predictive value (NPV) was 0.85). For Philips®-reported AHI_flow_, accuracy was low (AUC of 0.51 [0.43–0.60]) for a threshold of 1.4/h (sensitivity was 0.87 and specificity was 0.21, PPV was 0.17 and NPV) was 0.90. For 95th ResMed® nasal leaks, accuracy was low with an AUC of 0.62 [0.49–0.75] for a threshold of 18 L/min (sensitivity was 0.77 and specificity was 0.45, PPV was 0.19 and NPV was 0.92). For 95th ResMed® oronasal leaks, accuracy was low (AUC of 0.58 [0.45–0.72]) for a threshold of 15.6 L/min (sensitivity was 0.65 and specificity was 0.51, PPV was 0.22 and NPV was 0.87). The HLL Philips® ROC curve could not be created because of a Hosmer and Lemeshow positive test, indicating invalid (ROC) values. We did not generate AHI_flow_ and HLL ROC curves for the 8 adherent patients treated with Fisher & Paykel® devices.

## Discussion

The 2013 American Thoracic Society statement [3] on the CPAP device tracking systems underlined the absence of standards for scoring flow signals, or measuring mask leaks, as well as the non-existence of standards on how to use these data in order to improve outcomes. Our analysis suggests that in long-term CPAP-treated patients, the 2013 ATS statement proposed thresholds for HLLs and AHI_flow_ are not associated with non-adherence. In addition, we failed to find statistically satisfactory AHI_flow_ and HLL thresholds for predicting non-adherence.

### AHI_flow_ thresholds

As underlined by the 2013 ATS statement, AHI_flow_ is not a true surrogate of AHI measured by polysomnography (AHI_PSG_). Indeed, previous studies have reported that AHI_flow_ was not always correlated or concordant with AHI_PSG_ and major differences exist between manufacturer definitions of the residual events [4, 5]. In this regard, different ROC-determined AHI_flow_ thresholds were found for different machines (as can be expected, considering that device manufacturers all use different event definitions [3]). Similarly, in short-term treated patients, Valentin et al. reported that Philips®-reported AHI_flow_ during the first week of treatment was associated with lower adherence to CPAP therapy at 5 weeks of treatment, but the authors were unable to propose an AHI_flow_ threshold [6].

### Leak thresholds

Our long-term study agrees with two other short-term studies focused on leaks. Valentin et al. demonstrated that device-reported leaks during the first week of treatment were slightly associated with lower adherence to CPAP therapy at 5 weeks of treatment (a threshold-adjusted leak-level of 4.9 L/min/cm H_2_O was associated with a sensitivity of 0.62 and specificity of 0.65 for discriminating adherent and non-adherent patients) [6]. Baltzan et al. reported (using a manual score of device-reported leaks with a cut-off of 20 l/min of unintentional leaks and patterns of continuous leaks or serrated leaks) that the highest quartile of percentage time in continuous leaks may be linked to adherence during the first 3 months of treatment (but the relationship did not reach statistical significance) [7]. The aetiology of leaks is also an important issue, and more attention should be given to mouth leaks, as recommended by the 2013 ATS statement [3]. Bachour et al. have reported that mouth breathing patients were less adherent to CPAP-treatment at 3 months [8]. In this regard, the fact that the present study indicates that the mouth dryness VAS score is associated with lower adherence during univariable analysis is quite interesting. Mouth dryness may potentially be the consequence of mouth leaks, although we cannot overcome other confounding factors in our study (medical prescriptions and co-morbidities) [9] that help explain the absence of significance at the multivariable level. A VAS score is not sufficient for the accurate evaluation of mouth opening and new tools are required. Recently, the suitability of a mandibular movement sensor for evaluating mouth opening effects on unintentional leaks was demonstrated [10] and may respond to this need [11].

### Study limits

Beyond these negative results, it is important to remember that our population was treated on a long-term basis and our conclusions may not be applicable to short-term situations. For long-term patients, in contrast with absolute-value thresholds, the percentage-change may be of greater interest [12]. However, our study design did not enable us to test this hypothesis.

## Conclusions

Six years after the 2013 ATS statement and during a time when telemedicine is growing, our data suggest that before proceeding with remote monitoring initiatives, it is necessary to validate the diagnostic potential of data generated by CPAP tracking systems before they are implicated in a decision making process.

## Data Availability

The datasets used and/or analyzed during the current study are available from the corresponding author on reasonable request.

## Abbreviations

AHI_flow_: Residual Apnea–Hypopnea-Index
ATS: American Thoracic Society
AUC: Area Under Curve
BMI: Body Mass Index
CPAP: Continuous Positive Airway Pressure
EQ-5D-3 L: Euroqol 5 Dimensions 3 level version
ESS: Epworth Sleepiness Scale
HLLs: High Large Leaks
VAS: Visual Analog Scale
